# Effect of Solution-to-Binder Ratio and Molarity on Volume Changes in Slag Binder Activated by Sodium Hydroxide at Early Age

**DOI:** 10.3390/ma17133308

**Published:** 2024-07-04

**Authors:** Maïté Lacante, Brice Delsaute, Stéphanie Staquet

**Affiliations:** BATir Department (LGC), Université libre de Bruxelles, 1050 Brussels, Belgium; brice.delsaute@ulb.be (B.D.); stephanie.staquet@ulb.be (S.S.)

**Keywords:** alkali-activated blast-furnace slag, sodium hydroxide, autogenous strain, coefficient of thermal expansion, isothermal calorimetry, internal relative humidity

## Abstract

This research investigates the impact of solution concentration and solution-to-binder ratio (S/B) on the volume changes in alkali-activated slags with sodium hydroxide at 20 °C. Autogenous and thermal strains are monitored with a customized testing device in which thermal variations are controlled. Consequently, both the autogenous strain and coefficient of thermal expansion (CTE) are determined. Heat flow and internal relative humidity (IRH) are also monitored in parallel, making this research a multifaceted study. The magnitudes of autogenous strain and CTE are higher than those of ordinary Portland cement paste. Decreasing the solution concentration or S/B generally decreases the autogenous strain (swelling and shrinkage) and the CTE. The shrinkage amounted to 87 to 1981 µm/m, while the swelling reached between 27 and 295 µm/m and was only present in half of the compositions. The amplitude of the CTE, which increases up to 55 µm/m/°C for some compositions while the CTE of OPC remains between 20 and 25 µm/m/°C, can be explained by the high CTE of the solution in comparison with water. The IRH of paste cannot explain the autogenous strain’s development alone. Increasing S/B eliminates the self-desiccation-related decrease.

## 1. Introduction

The traditional component of concrete is ordinary Portland cement (OPC). Regrettably, its worldwide annual production is accountable for 5% to 8% of anthropogenic carbon dioxide (CO_2_) emissions [1,2,3]. Promising and vital substitutes to OPC are alkali-activated binders, which are clinker-free. An alkali-activated material (AAM) is formed when an alkaline solution is mixed with a precursor. Various solutions and precursors can be used for this purpose, including sodium hydroxide, potassium hydroxide, sodium silicate, or sodium sulfate as activators and blast-furnace slag, steel slag, or fly ash as precursors. A portion of the binders can be substituted with metakaolin or limestone filler [4]. Their associated CO_2_ footprint can be up to 80% less than that of OPC while offering the same potential. It is important that those materials offer comparable performances and characteristics, considering that concrete is the second most commonly used material globally, after water [1,5].

**Figure 1:**
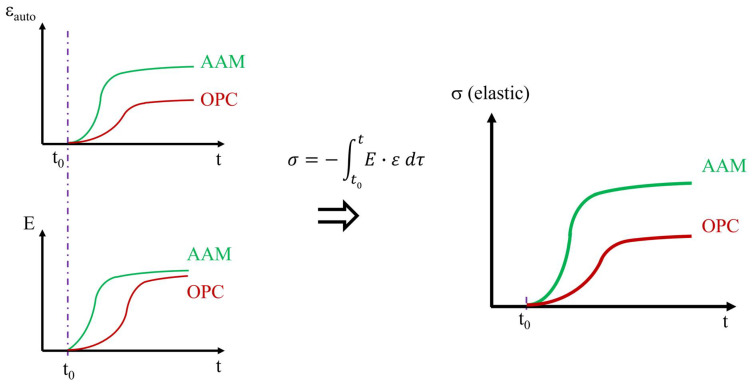
Stress development in AAM and OPC, *t*_0_ is the final setting time.

Many compositions studied in the literature have demonstrated numerous advantages, such as high early strength [6], good fire resistance [7], and improved resistance to chemical attack [8]. However, alkali-activated materials often suffer from significant shrinkage. This is because, unlike OPC concrete, which typically sets in four to eight hours for a water-to-cement ratio (W/C) between 0.3 and 0.4 [9], alkali-activated materials usually set faster, depending on the alkali content [10]. AAMs exhibit higher shrinkage (autogenous and drying) on longer duration and develop faster E-modulus with similar magnitude (see Figure 1). Consequently, when the material is used in restrained conditions, it may lead to early-age cracking due to the tensile stress build-up. This must be avoided to ensure the durability of concrete structures [11,12,13,14].

Concrete is a material characterized by continuously evolving properties and undergoing volume changes over time. Therefore, it is important to study the behavior of the material and its early-age volume changes in both sealed and drying conditions. The former comprises autogenous and thermal deformations induced by the reaction processes and thermal changes, respectively [15]. The drying process is not studied in the frame of this research but has been investigated by Sirotti et al. [16] and Sirotti et al. [17].

Autogenous strain is a phenomenon occurring in the early stage in cement-based and alkali-activated materials. It can cause significant shrinkage, leading to cracks in the sample, which can damage the material and reduce its mechanical properties and durability. Autogenous strains encompass three different mechanisms: chemical shrinkage, swelling, and self-desiccation [11,18]. During the reaction process, the material undergoes hydration, resulting in a reduction in volume, known as chemical shrinkage or contraction of Le Chatelier. The volume of the products is smaller than that of the reagents [19,20]. The occurrence of swelling after setting has been reported by some authors [11,21], followed by subsequent shrinkage. However, the cause of the swelling is not yet fully understood, and several hypotheses have been proposed, such as the reabsorption of water by the OPC paste during the reaction and the volume of the hydration product (such as portlandite and ettringite) [21,22,23]. In AAM containing blast-furnace slag, the swelling is more likely to be caused by crystalline reaction products (hydrotalcite group minerals), whose amounts are limited and which are less expansive than ettringite [24]. After the consumption of the solution in the pores, a depression occurs in the solid skeleton, leading to a global contraction of the paste. This phenomenon is the shrinkage due to self-desiccation. Three different mechanisms contribute to self-desiccation: capillary tension, disjoining pressure, and surface tension [11,25]. During the reaction, the pores in the material become filled with a solution that is eventually consumed, causing some pores to become voids and creating liquid-vapour menisci in others. This generates capillary tension in the pores and decreases the internal relative humidity, leading to shrinkage [9,26]. In areas where absorption is hindered, a disjoining pressure is created, which increases the separation of adjacent surfaces, if the thickness of the absorption layer is two times greater than the distance between the two solid surfaces. Decreasing the internal relative humidity reduces this disjoining pressure and causes shrinkage [11,27]. Finally, surface tension depends on the internal relative humidity and mainly occurs in materials with a large internal surface, such as during the reaction. When the internal relative humidity increases, the absorbed water decreases the surface tension. During hydration, the thickness of the absorption layer decreases, increasing the surface tension [28]. However, the surface tension remains relatively low when the internal relative humidity is high [29]. The magnitude of the self-desiccation observed in AAM is much higher than in OPC because the pore structure of AAM is denser, the pore solution’s surface tension is larger, and the degree of saturation is higher, resulting in a higher capillary pressure. Moreover, deformability also plays a role due to the high viscoelastic nature of the C-A-S-H gel [24]. In addition to self-desiccation due to capillary pressure, other causes have been proposed for slag-based AAM. The shrinkage might be induced by polymerization. In fact, as the formation of the solid network happens, a reaction of poly-condensation occurs between two adjacent gel units resulting in shorter distances between the solid particles [25]. Another explanation is the reduction in the repulsive steric-hydration force because of a decrease in the ions concentration in the pore solution. During that time, the opposite attractive force acting between the gel particles remains, resulting in shrinkage [30].

Autogenous shrinkage for OPC can reach up to 1100 µm/m when its W/C is between 0.3 and 0.4 [9], while for AAM, it remains between 3000 and 8000 µm/m depending on the different parameters that characterize the AAM, such as alkali dosage and silicate modulus [31]. Ballekere Kumarappa et al. [32] and Uppalapati [25] have demonstrated that although the autogenous strain of AAM is reduced at mortar scale (between 1500 and 5000 µm/m) due to the restraint induced by the sand and the low E-modulus at early age when the autogenous strain develops, it remains substantially higher than that of OPC (about 500 µm/m).

To investigate and quantify the thermal strains of cementitious materials, the coefficient of thermal expansion (CTE) is determined. Four different stages are identified. In the initial stage, the paste is in a plastic phase, exhibiting a relatively high CTE influenced by water. Transitioning to the second stage, the CTE experiences a rapid decline as the paste begins to set, reaching a minimum value corresponding to that of the solid skeleton. Progressing into the third stage, the CTE rises again due to a reduction in the internal humidity of the paste. Lastly, the fourth stage is characterized by a relatively constant CTE [11]. Understanding these stages is crucial for various applications, underscoring the significance of this study in the field. Moreover, research on the CTE for AAM is limited, especially at the early age. While Sellevold et al. [33] have demonstrated that the CTE is time-dependent, ranging between 15 and 30 µm/m/°C for OPC with W/C between 0.40 and 0.55 [34], only a few studies have examined the CTE of AAM due to the difficulty of determining it over time. Ma et al. [35] have reported a CTE of 16.6 µm/m/°C for AAM concrete, while OPC concrete typically exhibits a CTE between 8 and 12 µm/m/°C [36,37].

Previous research has focused on a blended system consisting of blast-furnace slag and fly ash activated with sodium hydroxide and sodium silicate [38]. However, the mechanisms behind the observed volume changes were not fully understood. Therefore, the main objective of this research is to gain a better understanding of the volume changes in a simpler system composed of blast-furnace slag activated by sodium hydroxide. The addition of slag increases the formation of N,C-A-S-H gels and decreases the porosity which decreases the Na^+^ and Si^4+^ leaching potential. Consequently, the diffusion of alkali ions is decreased in slag-based systems compared with fly-ash-based systems. This is beneficial to protect the reinforcing steel from corrosion because the concentration of leached alkali ions is reduced [39].

As observed by Li et al. [24], AAMs are very sensitive to the components and their content. Even minor modifications in a single parameter can have a substantial impact on the results. This study investigates the sensitivity of the concentration of the activator and the solution-to-binder ratio on the volume changes in blast-furnace slag activated with sodium hydroxide. The volume changes under investigation include free deformation without drying: autogenous strain $\epsilon_{auto}$ and more interestingly, thermal strain $\epsilon_{th}$. The latter is defined based on the development of the evolution of the coefficient of thermal expansion. This is a crucial parameter for the thermal characterization of these types of materials and is quite rarely investigated due the complexity of its measurement. To better investigate the development of the autogenous strain and the coefficient of thermal expansion, the thermal behavior of alkali-activated materials, including their cumulative released heat Q and heat flow, along with their internal relative humidity IRH, was monitored in parallel as well [14], as shown by Equation (1). This study investigates various aspects concurrently, exploring parallel relationships within the research framework. No study known to the authors combines and studies all these parameters together.

(1) $$\epsilon_{auto}=f\left(IRH\right)\wedge \epsilon_{th}=f\left(CTE,Q\right)$$

## 2. Materials and Methods

### 2.1. Material Characterization

This study considers pastes obtained through the alkali-activation of blast-furnace slag (BFS) with sodium hydroxide, see Table 1 for the chemical composition. Details regarding the particle size distribution can be found in Dai et al. [40]. The BFS has a Blaine fineness of 516 m^2^/kg and a density of 2.92 g/cm^3^. The activating solutions were prepared using a pellet form NaOH with 97% purity. All materials and solutions were cured at 20 °C in sealed containers for at least 24 h before the mixing [41]. After the mixing, the samples are sealed and cured at 20 °C within 45 min at most, depending on the workability of the composition.

The activating solution is sodium hydroxide with three different molar concentrations: 0.5 M, 2 M, and 8 M.

### 2.2. Pastes Design

This study aims to assess and quantify the influence of each constituent of the pastes on volume changes during the early stages. To achieve this, six different pastes were analyzed, each with variations in the concentration of the activating solution and the solution-to-binder mass ratio (S/B) where “binder” is defined as the BFS content, as detailed in Table 2.

The investigation of S/B is motivated by the fact that the W/C ratio is a primary internal parameter impacting the development of the autogenous strain in OPC pastes [42]. The S/B ratio is equal to 0.5 and 0.8 due to the observation that lower S/B compositions tend to exhibit very poor workability, particularly when slag is the sole binder [43], while higher S/B ratios increase the risk of bleeding. Furthermore, the concentration of the solution plays a role in the reaction process and consequently affects the autogenous deformation [44]. Therefore, the molar concentration of the NaOH activator will be investigated at levels of 0.5, 2, and 8 molar. This way, a range of interesting and realistic concentrations in this field are studied while maintaining a factor of 4 between each proposed concentration. Under 0.5 M, the reaction will not be sufficient. Above 8 M, the solution heats up too much and it becomes difficult to obtain a transparent and, thus, homogeneous solution. This classic approach facilitates the comparison with the literature.

The pastes were prepared in accordance with the procedures outlined in the European Standard EN 196-1:2016 [45]. The control of the quality of the slag to avoid degradation and the control of the proper mixing are performed by conducting a slump test each test and isothermal calorimetry regularly. The results were compared with those of OPC pastes with water-to-cement ratios (W/C) of 0.3 and 0.5 [46]. Within this range, the compositions are stable. Specifically, a higher W/C results in a paste that is too liquid, whereas the addition of admixtures becomes necessary when the W/C is lower.

## 3. Methods and Devices

### 3.1. Workability

The workability of the paste is assessed using the ASTM cone (ASTM C230 [47]). Within 5 min after mixing, the workability is evaluated by measuring the average diameter of the paste disc, with a measuring error of 5 mm. The mean slump diameter obtained from different tests, along with the standard deviation, will be reported in the results. For S05M05, S08M2, and S08M8, 4 measurements were performed, while for S05M2, S05M8, and S08M8, 6 measurements were carried out. As the hardening process advances, both the diameter and workability decrease over time. However, as the primary focus of this work does not encompass mechanical properties, a flowability test over time has not been carried out. This method is employed to ensure the repeatability of the mixing throughout the entirety of the testing campaign.

### 3.2. Compressive Strength

The compressive strength of the pastes is evaluated by testing cubes (50 mm side). The tests were conducted on a hydraulic Galdabini press of 600 kN with a sensitivity of 1kN following the ASTM C109 standard [48], which stipulates that the loading rate should range between 900 and 1800 N/s. Moreover, this rate must be obtained within the first half of the anticipated maximum load and no adjustments are allowed later. The paste is poured into molds with the right dimensions. After being vibrated to remove the air bubbles, the paste-filled molds are immediately sealed with plastic sheets and stored in the climatic chamber at 20 °C. As per previous works [41,46], two cubes are tested for each composition at each age (i.e., 1, 3, 7, and 28 days). Thanks to the great repeatability, no additional tests were conducted as the range between specimens was less than 7.6%, as required by the ASTM C109 standard [48].

### 3.3. Setting Times

The setting times of the pastes were determined using the Vicat apparatus according to the EN 196-3:2016 standard [49]. The initial setting time is defined as the time elapsed from when the activating solution is added to the binder (time zero) until the distance between the base plate and the needle is (6 ± 3) mm. Conversely, the final setting time is defined as the time elapsed from when the activating solution is added to the binder until the needle only penetrates 0.5 mm (or less) into the specimen. In OPC-based materials, the initial set marks the point where the material loses its workability and begins to stiffen [43,50], while the final set marks the time needed for the material to completely lose its plasticity or stiffen without significantly developing its strength [51]. The initial and final setting times will be reported to the nearest 5 min, in accordance with the European standard EN 196-3:2016.

Naqi et al. [41] have observed limitations in utilizing the Vicat method for determining the initial and final setting times in AAM, particularly in adequately describing the transition from a fluid to a solid state, which can be unpredictable in the case of AAM [41]. Therefore, the results obtained from the Vicat criteria will be compared with the setting times determined by using the isothermal calorimetry [50] and autogenous strain [52], see Section 4.

### 3.4. Cumulative Heat, Heat Flow, and Activation Energy

Heat release is monitored by means of isothermal calorimetry to follow the progression of the reaction process of the material [53]. For this purpose, the TAM Air calorimeter is used [54]. The device used in this study has eight channels that can be used simultaneously at the same temperature. Each channel contains two spots, one for the sample (ampoule filled with about 7.5 g of material) and one for a reference (sand in this case), each equipped with its own heat flow sensor. The heat flow from the tested sample is directly compared with that of the inert reference thanks to this twin configuration, thus reading noise and increasing measuring stability. Two samples of each composition are monitored for a duration of two weeks. After this age, the heat flow is generally too low to be accurately monitored [46,55].

### 3.5. Internal Relative Humidity

**Figure 2:**
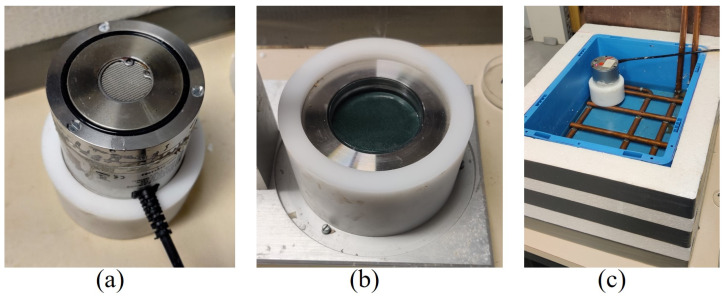
IRH set-up: (**a**) IRH sensor; (**b**) sample in holder; (**c**) sealed box.

The internal relative humidity (IRH) is measured using HC2-AW water activity probes, as illustrated in Figure 2a, which are calibrated with different salt solutions with known relative humidities. The pastes are cast in plastic cylinders (Ø43 mm), sealed, and placed in a thermostatically controlled box maintained at (20 ± 0.1) °C, as depicted in Figure 2b,c. The measuring accuracy of the probes is ±1%.

### 3.6. Autogenous Strain and Coefficient of Thermal Expansion

**Figure 3:**
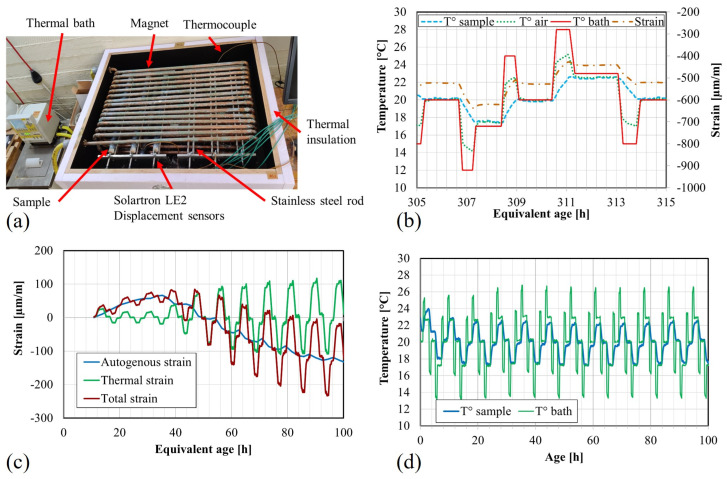
AutoShrink explanation: (**a**) AutoShrink device developed at ULB [38] (modified); (**b**) example of temperature variations [38]; (**c**) example of measured strains; (**d**) example of temperature history.

The AutoShrink digital dilatometer, manufactured by Germann Instruments [56] is based on the method initially proposed by Jensen and Hansen [57]. Its primary purpose is to monitor the (linear) autogenous strain of cementitious materials cured under sealed conditions during the hardening process. Special corrugated plastic molds (Ø30 mm) prevent moisture loss while allowing the specimen to shrink without being restrained longitudinally, thereby meeting the sealed conditions. These 400 mm long molds are fixed at one end to the rigid frame using a magnet, while a digital gauge records the displacement of the sample with respect to the frame at the other end. The deformation of two samples is monitored, in addition to the internal temperature of a third sample. Data acquisition for the displacement and temperature occurs every minute. Distinguishing the thermal strain from the autogenous strain can be difficult because both parameters evolve at the same time. Therefore, the customized AutoShrink at ULB (see Figure 3a) is equipped with a thermal regulation around the rigid frame to apply repeated temperature variations (variations of ±3 °C around the curing temperature of 20 °C, as depicted in Figure 3b,d). These controlled temperature variations allow for determining the evolution of the coefficient of thermal expansion (CTE) by monitoring the resulting thermal strain. In fact, the CTE can be considered constant with respect to the temperature for such small thermal variations [58]. Additionally, effective thermal regulation ensures no remaining thermal gradients due to heat release. However, it is noted that CTE is age- and maturity-dependent, as discussed in Section 1. Since the repeated temperature variations are applied to the entire set-up, the rigid frame undergoes thermal deformation as well. Consequently, the temperature inside the AutoShrink is recorded in addition to the internal temperature of the third sample. Thanks to the calibration of the setup, the coefficient of thermal expansion of the device was determined previously [38]. Subsequently, the thermal strains of the set-up can be subtracted from the raw results to only have the (total) strains related to the paste.

The total strain $\epsilon_{tot}$ of the sample is determined during the test (see Figure 3c), meaning that the thermal strain and the autogenous strain have to be decoupled (see Equation (2), where $\epsilon_{auto}$: autogenous strain [µm/m]; α: coefficient of thermal expansion of the material [µm/m/°C]; ΔT: temperature variation [°C]) [38,46,58].

(2) $$\epsilon_{tot}=\epsilon_{auto}+\alpha \cdot \Delta T$$

The decoupling procedure has been established by Delsaute and Staquet [58]. To implement the procedure, a sample cured at 20 °C (without any external thermal variations) is considered by determining a fictitious strain evolution based on a cubic interpolation fitted to the measured strain when the applied temperature is 20 °C, at the end of the cycle interval (to ensure that no remaining thermal gradient exists in the material). The thermal strain is obtained by subtracting the fictitious strain (index 1) from the total measured strain (index 2), as shown in Equation (3). Once the CTE evolution is computed based on the thermal strain, the autogenous strain can be calculated (Equation (2)). Therefore, CTE and the autogenous strains are computed about every 2 h.

(3) $$\alpha =\frac{\Delta \epsilon_{tot,1}-\Delta \epsilon_{tot,2}}{\Delta T_{1}-\Delta T_{2}}$$

## 4. Results and Discussion

### 4.1. Workability

**Figure 4:**
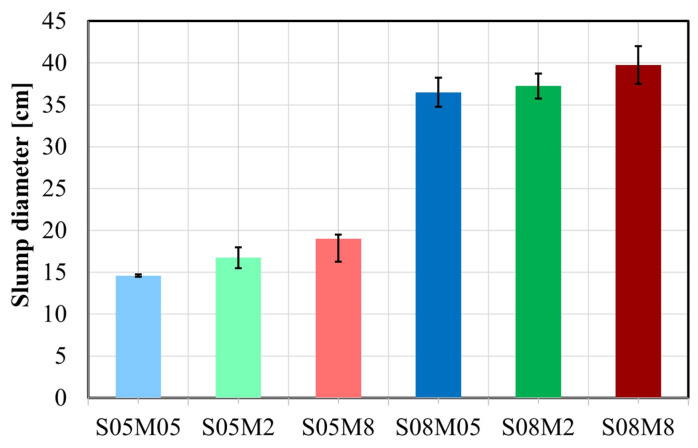
Workability of AAS pastes.

The workability of the alkali-activated slag (AAS) pastes is significantly influenced by their S/B ratio, as shown in Figure 4. A higher S/B ratio results in a more fluid paste due to the increased addition of solution, which enhances the workability, similarly to OPC pastes [59]. Furthermore, the concentration of the alkali solution has a minor impact: increasing the alkali content leads to higher particle dispersion [60], resulting in increased slump flow. The impact of the molarity is more pronounced for compositions with low S/B. However, this effect of the S/B ratio is considerably more significant than that of the alkali concentration.

### 4.2. Compressive Strength

**Figure 5:**
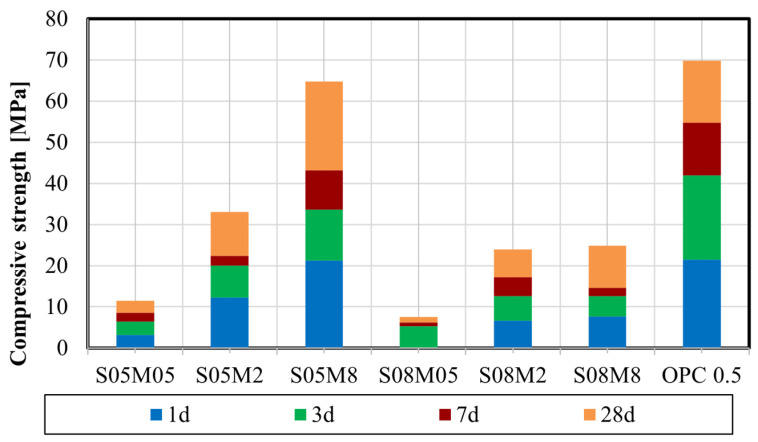
Compressive strength of AAS pastes.

Figure 5 shows the compressive strength of AAS pastes at the age of 1 day, 3 days, 7 days, and 28 days. The compressive strength of composition S08M05 is considered as zero at 1 day of age, as both samples broke during the preloading phase (<2 kN). The concentration of the alkaline solution significantly influences the compressive strength. Increasing the concentration of the alkaline solution leads to higher compressive strength in alkali-activated materials. The quantity of activator used in the mix plays a crucial role in promoting the formation of C-A-S-H gel, which is responsible for providing strength to alkali-activated materials. Therefore, a higher amount of activator results in the formation of a greater quantity of C-A-S-H gel, thereby contributing to the increased strength [61].

In addition, the S/B ratio also has a decreasing impact on the compressive strength, as it relates to the quantity of water in the mix. The addition of water induces a lower solid volume fraction which decelerates the formation of the reaction products. As a result, the microstructure is less dense and the material strength is lower [41].

The highest compressive strength results were obtained for S05M8, whose results were similar to the compressive strength obtained for OPC paste with a W/C of 0.5.

### 4.3. Setting Times

**Figure 6:**
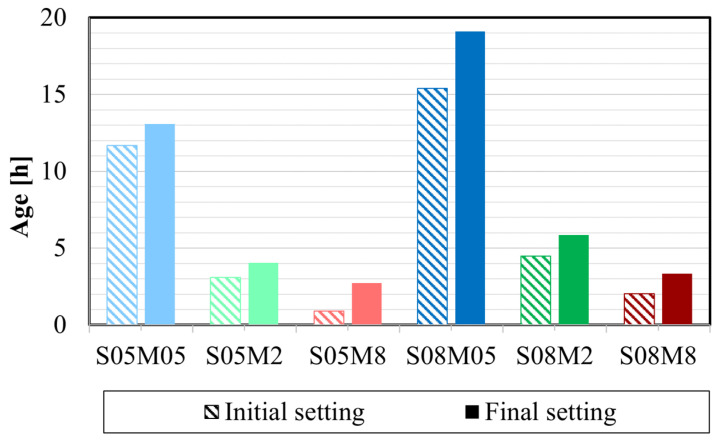
Setting times of AAS pastes determined with the Vicat method.

As depicted in Figure 6, S/B exhibits an increasing effect on the setting times, similar to the effect of W/C on the setting of OPC [59]. However, an increase in the concentration of the alkaline solution decreases the setting times [62]. In fact, Sun et al. [60] have reported that the release of more ions in the solution, attributed to the higher molar concentration of the alkaline solution, enhances polymerization and increases the reaction rate, thereby accelerating the setting.

### 4.4. Isothermal Calorimetry

#### 4.4.1. Heat Flow and Cumulative Heat

**Figure 7:**
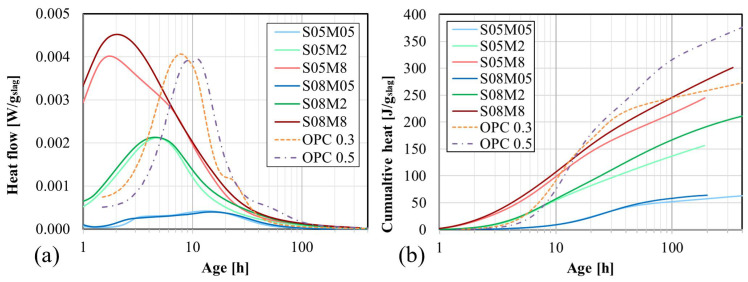
Isothermal calorimetry of AAS pastes: (**a**) heat flow and (**b**) cumulative heat normalized by the mass of slag.

As it can be seen from Figure 7a,b, the reaction process of AAM paste is exothermic. The evolution of the heat flow is usually characterized by two peaks: The first peak, related to the early dissolution of the slag, cannot be observed clearly because it occurs very quickly after mixing, within just a few minutes. This is the time needed to transfer the paste into the isothermal calorimeter. Moreover, some time is also needed to stabilize the sample at the exact curing temperature. Consequently, the presented results only start after one hour. To quantify the cumulative heat lost by this delay, an in situ test was conducted: a maximum of 11 J/g at 1 h was measured for a composition similar to S05M8. Considering that 237 J/g is measured after 7 days (168 h), the initial cumulative heat is therefore deemed negligible, as it represents less than 5% of the cumulative heat at 7 days. In addition, Dai et al. [40] demonstrated that the cumulative heat resulting from the first peak is relatively small with respect to the cumulative heat generated later. Despite its high amplitude, the first peak happens very quickly, leading to its insignificant contribution to the cumulative heat in comparison with the second peak, which lasts much longer. The second peak corresponds to the formation of the reaction products, specifically calcium silicate hydrate through silicate hydration [63]. Figure 7a,b indicates that an increase in both the concentration of the alkaline solution and S/B has an increasing effect on the heat flow and cumulative heat, respectively.

The main influencing factor is the concentration of the alkaline solution. A higher concentration accelerates the reaction, causing the heat flow to reach its maximum faster. This is attributed to the higher amount of OH^−^, which improves the dissolution of the slag particles, therefore contributing to the acceleration of the reaction. In addition, the induction period is reduced [64].

At an early age, the S/B ratio does not have a significant influence. Only after a few hours does the S/B ratio begin to impact the heat release, which increases with a higher S/B ratio. This trend can be observed in the cumulative heat results, as depicted in Figure 7b. In fact, for the same concentration, both S/B curves initially remain superimposed. After some time, the curves start to separate due to the different S/B ratios, as the alkali content decreases faster with time for a lower S/B ratio. Regarding the heat flow results, an increase in S/B does not change the amplitude of the second peak significantly. However, the second peak starts earlier and is prolonged due to the direct monomers polymerization [65].

Next, the results are compared with those of OPC paste. Concerning the heat flow, the second peak of OPC appears a few hours later than that of the AAS paste and is comparable in magnitude to the 8 M pastes. The cumulative heat is also postponed with respect to the AAS paste but ultimately reaches a higher value.

**Figure 8:**
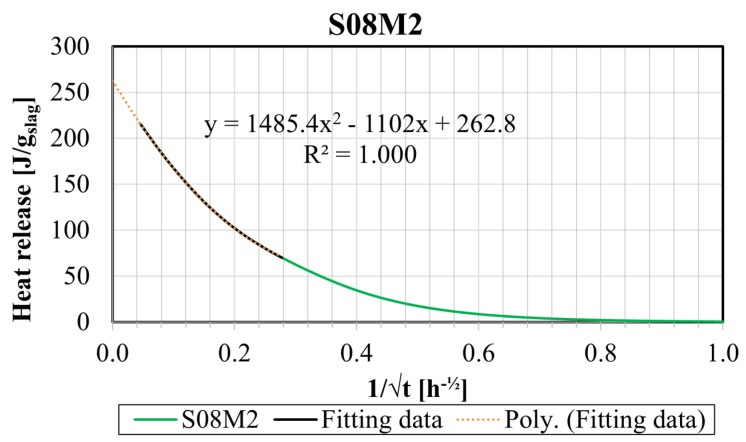
Extrapolation of the ultimate heat Q_∞_ for S08M2.

Afterward, the ultimate heat release, Q_∞_, was determined by extrapolating the cumulative heat Q(t) curve. Here, the ultimate heat release is considered as the heat released after an infinite time for this composition in these testing conditions, in other words, the maximal heat release that the composition can reach. The cumulative heat was plotted against the inverse of the square root of the age of the composition, and the size of the extrapolation interval was chosen to be as large as possible while keeping the R^2^ value of the fitting as close to 1 as feasible. The extrapolation interval and R^2^ are presented in Table 3. Q_∞_ was considered as the intersection of the extrapolation curve and the vertical axis [66]. Although a linear extrapolation would be the most adequate method to determine this parameter, it did not provide a satisfactory fit for all compositions. Therefore, a second-order polynomial extrapolation was used for all six compositions. As an example, the extrapolation for the S08M2 composition can be found in Figure 8.

**Figure 9:**
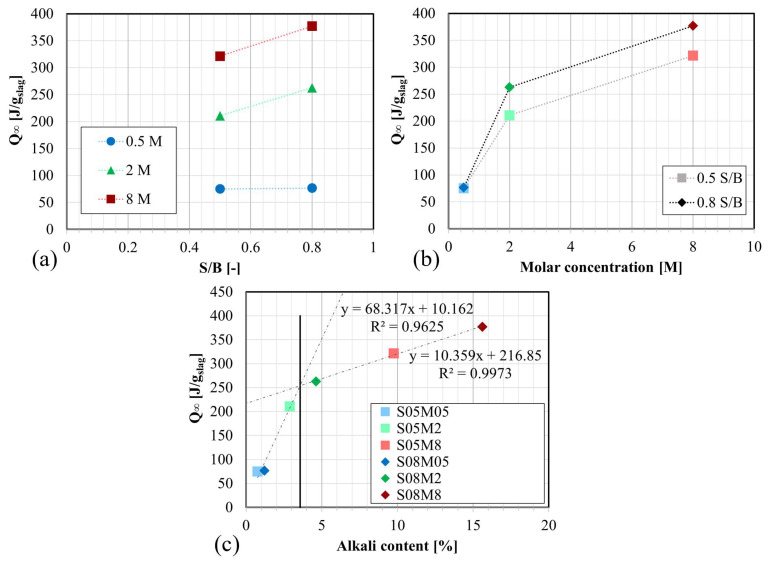
Ultimate heat of AAS pastes: (**a**) as a function of S/B; (**b**) as a function of the molar concentration; (**c**) as a function of the alkali content.

The results for each composition are provided in Table 3 and are plotted as a function of S/B and M for each composition in Figure 9a,b. The ultimate heat increases when the alkaline concentration increases. The S/B ratio has the same effect.

Finally, the ultimate heat can be represented as a function of the alkali content, see Figure 9c. The alkali content represents the Na_2_O mass content of the paste and is calculated based on the S/B ratio of the paste, the molar concentration, and the density of the alkaline solution. Two lines can be drawn: one comprising S05M05, S08M05, and S05M2, and another comprising S08M2, S05M8, and S08M8. The optimum alkali content in terms of ultimate heat can be identified as being the intersection of both lines. For AAS paste, this is 3.57%. The optimum alkali content indicates the maximum alkali content beyond which a smaller increase in ultimate heat is obtained for the same increase in alkali content. Notably, the first line has a slope of 68.32 J/g, while the slope of the second line is 10.36 J/g, which is much smaller.

The ultimate heat allows for determining the degree of reaction α(t) defined by Equation (4), which will be used to compare further results.

(4) $$\alpha \left(t\right)=\frac{Q(t)}{Q_{\infty }}$$

#### 4.4.2. Setting Times Defined from Isothermal Calorimetry

**Figure 10:**
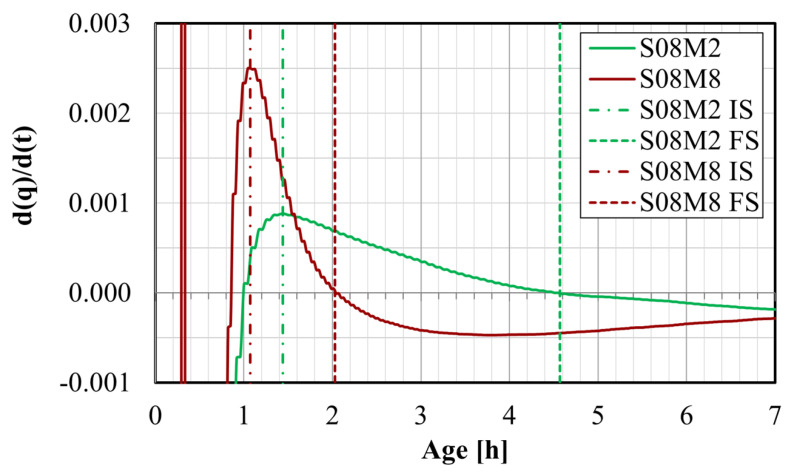
First derivative of the heat flow of S08M2 and S08M8.

Finally, the setting times were evaluated based on the first derivative of the heat flow results. According to Hu et al. [67], the initial set (IS) corresponds to the time at which the first derivative is at its maximum. This corresponds to the moment where the heat generation rate is the fastest. On the other hand, the final set (FS) occurs when the first derivative is zero, indicating the highest rate of reaction, which decreases afterward. The procedure for determining the setting times for S08M2 and for S08M8 is represented in Figure 10.

The initial setting times (IS) and final setting times (FS) of each composition obtained through Vicat testing and through the heat flow’s first derivative method are shown in Table 4. The best agreement between methods is obtained for the final setting times of the S05 compositions where the obtained ages are very similar. The results for S08M05 exhibit the largest disparity between both methods. Notably, both methods yield comparable results for compositions with a low S/B and a high concentration (i.e., S05M8). However, as the concentration of the alkaline solution is reduced or when the S/B ratio is increased, the results diverge significantly.

#### 4.4.3. Apparent Activation Energy

The apparent activation energy (E_a_) was determined using the Arrhenius law through isothermal calorimetry conducted at 10, 20, and 30 °C [38]; see Table 5. An average of 75.50 (±8.13) kJ/mol was obtained for AAS pastes. Previous literature studies obtained 75.2 (±6.7) kJ/mol [68] and 73.2 (±2.8) kJ/mol [38] for alkali-activated materials.

Subsequently, the apparent activation energy was used to compute the equivalent age t_eq_ using Equation (5), where *T(t)* is the temperature at time *t* in Kelvin and *T_ref_* is the reference temperature, which was 293.15 K (20 °C) [69,70].

(5) $$t_{eq}\left(t\right)=\int_{0}^{t}exp\left(-\frac{E_{a}}{R}\cdot \left(\frac{1}{T(\tau )}-\frac{1}{T_{ref}}\right)\right)d\tau$$

### 4.5. Autogenous Strain

**Figure 11:**
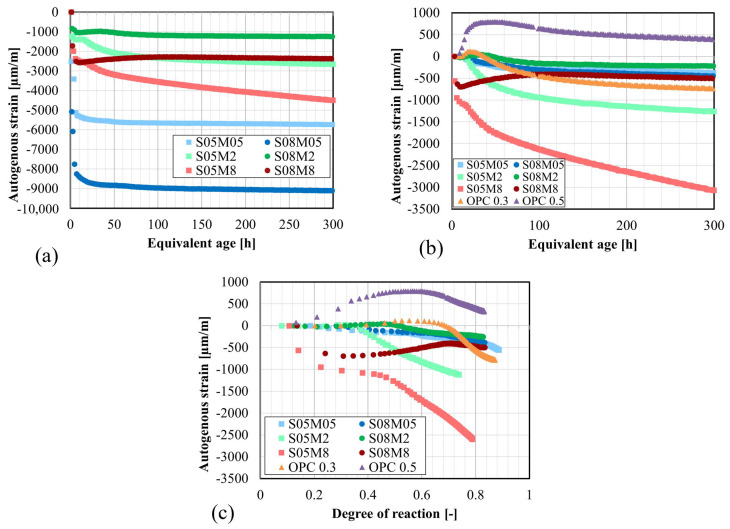
Autogenous strain of AAS pastes: (**a**) as a function of the equivalent age, t_0_ = t_test start_; (**b**) as a function of the equivalent age, t_0_ = t_FS,Vicat_; (**c**) as a function of the degree of reaction, determined with Equation (4), t_0_ = t_FS,Vicat_.

Figure 11a depicts the autogenous strain of AAS pastes, where time zero indicates the start of the test. As expected, the development of the autogenous strain exhibits three distinct stages. Initially, all compositions display significant shrinkage ranging from 1000 to 8500 µm/m, attributed to the chemical shrinkage of the paste as mentioned previously [25]. Compositions prepared with the 0.5 M solution exhibit higher initial shrinkage (approximately 5100 µm/m for S05M05 and 8400 µm/m for S08M05), while those made with 2 M and 8 M solution show less shrinkage: 1400 µm/m for S05M2, 1000 µm/m for S08M2, 2500 µm/m for S05M8, and 2500 µm/m for S08M8. However, it should be noted that the zero time of the test represents the time at which the acquisition started, which is not immediately after the mixing since the sample preparation takes between 45 and 75 min depending on the workability of the paste. Additionally, as seen beforehand, the Vicat setting times significantly depend on the studied properties (M and S/B). Therefore, some compositions such as S05M8 and S08M8 may already be close to their final set according to the Vicat procedure, whereas compositions like S08M05 may not have reached the initial set, potentially explaining the high shrinkage. The second stage, corresponding to swelling, is not fully evident for all compositions. When a 0.5 M solution is used, no apparent swelling is observed. However, the end of the shrinkage is clearly distinguishable, particularly when S/B is 0.5. S05M2, S08M2, and S08M8 exhibit swelling, respectively, 27 µm/m over 4.3 h, 71 µm/m over 25.9 h, and 295 µm/m over 106 h. Lastly, self-desiccation shrinkage occurs, characterized by a much slower rate compared with the initial shrinkage. At 300 h, it amounts to about 380 and 450 µm/m for S05M05 and S08M05, 1278 and 269 µm/m for S05M2 and S08M2, and 1981 and 87 µm/m for S05M8 and S08M8. For S05M05, S08M05, and S05M8, determining the actual self-desiccation shrinkage was challenging as no apparent swelling was observed for these compositions.

Figure 11b,c displays the autogenous strain as a function of equivalent age and degree of reaction, respectively. In this analysis, the autogenous strain was set to zero based on the final setting times obtained through Vicat testing (t_FS, Vicat_) for each composition, following the common practice for OPC compositions [9,57]. This approach facilitates a more detailed examination of the behavior of each composition once the chemical shrinkage has ended.

Increasing the S/B ratio leads to more significant swelling in the paste. Notably, S08M8 shows significantly more swelling than the other compositions and for a longer duration in terms of age. Shrinkage becomes noticeable later in the reaction. Moreover, increasing S/B decreases both the initial shrinkage and the self-desiccation shrinkage.

Regarding the concentration of the alkaline solution, an increase in concentration leads to an increase in shrinkage due to self-desiccation. Its effect on swelling is more complex. At higher S/B, the concentration has an increasing effect on swelling. However, at lower S/B, it is more difficult to determine. For instance, S05M2 exhibits a slight apparent swelling, while S05M05 does not show any swelling. On the other hand, S05M8 seems to have both swelling and self-desiccation shrinkage occurring simultaneously, but the self-desiccation shrinkage is too significant for the swelling to prevail. This is supported by the observation that the shrinkage occurring later is higher than that of the other compositions and that S/B is low, indicating that the solution is mostly consumed.

When the concentration of the alkaline solution increases, the effect of increasing S/B on swelling is amplified. Conversely, decreasing S/B enhances shrinkage. Moreover, an increase in S/B appears to postpone the occurrence of shrinkage due to self-desiccation. Compared with OPC, some compositions exhibit more shrinkage than OPC pastes. C-A-S-H gels are more viscoelastic, translating in higher deformability of these materials, typically when capillary pressure occurs [24].

Compared with OPC, AAS paste exhibits less swelling. The magnitude of the swelling increases with higher W/C. In OPC, the swelling might be attributed to the formation of ettringite [21,30] or the increase in IRH resulting from the reabsorption of the solution [23]. Similar phenomena occur in AAM. Moreover, as explained in Section 4.7, the increase in IRH in AAM can be linked to the increase in the total ion concentration. However, swelling in alkali-activated slag might also result from the formation of reaction products (e.g., hydrotalcite group minerals) [18]. Since the formation of these crystals is limited and they expand less than ettringite, the swelling observed in alkali-activated materials is less significant than in OPC paste [24].

**Figure 12:**
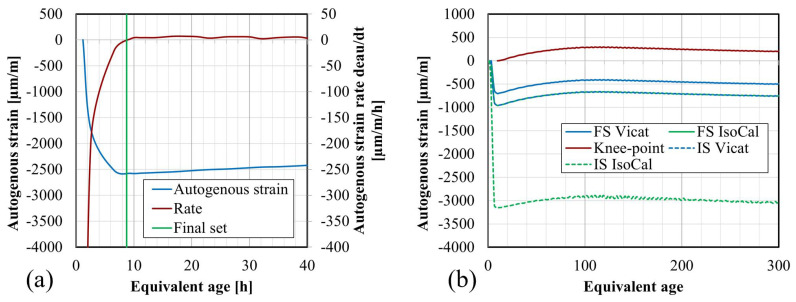
Application of the knee-point method and representation of different setting times to autogenous strain of S08M8 composition: (**a**) determination of final setting time with knee-point method (Rate of autogenous strain $d\epsilon_{au}/dt$); (**b**) autogenous strain of S08M8 initialized at the different obtained setting times: final set from Vicat, final set from isothermal calorimetry (heat flow), knee-point method, initial set from Vicat and initial set from isothermal calorimetry (heat flow).

Next, the setting times are determined based on the knee-point method; see Figure 12a for composition S08M8. This method consists in plotting the rate of autogenous strain against the age and identifying the point where the rate reaches zero, which corresponds to the transition between the solid state and the fluid state of the paste [52]. In cases where the rate never became positive, typically those that do not exhibit apparent swelling, the maximum value is taken as the setting time. The results can be found in Table 6.

For S05M05, S05M8, and S08M05, the setting times obtained using the knee-point method are significantly different from the other setting times. These discrepancies arise because no swelling occurs for these compositions, making it challenging to accurately determine the knee-point mathematically. In contrast, for the other three compositions, although the results differ, they are still closer together compared with the previous compositions.

**Figure 13:**
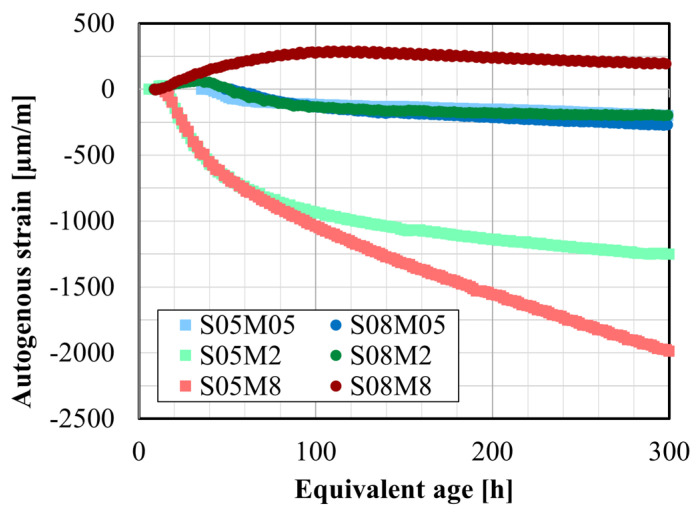
Autogenous strain of AAS pastes, initialized with the knee-point method.

In Figure 12b, the effect of various initialization times for S08M8 can be observed. Despite the knee-point method leading to an initialization at 9.17 h, compared with 3.33 h and 2.03 h for Vicat and isothermal calorimetry, respectively, the results appear consistent. This suggests that using the Vicat final setting time as time zero might not be the optimal choice for these materials, unlike for OPC where a relatively good agreement was found between the setting times defined with the Vicat device and the knee-point method [52]. However, determining the setting times based on other methods remains relatively challenging. Naqi et al. [41] have investigated and compared different methods to determine the setting behavior of AAM, including ultrasonic pulse velocity, slump flow, Vicat, heat flow, and compressive strength, reaching similar conclusions as in this study. Finally, the autogenous strains of all compositions initialized with the knee-point method can be found in Figure 13.

### 4.6. Coefficient of Thermal Expansion

**Figure 14:**
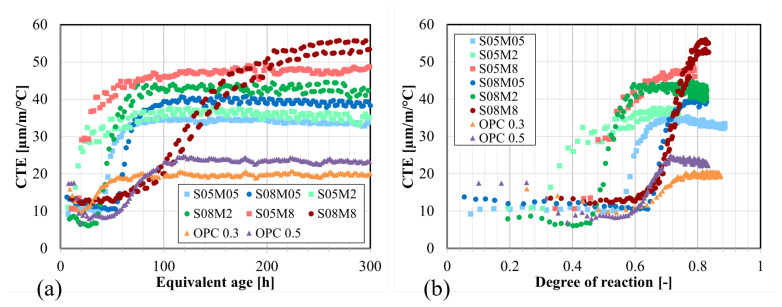
Coefficient of thermal expansion of AAS pastes: (**a**) as a function of the age; (**b**) as a function of the degree of reaction.

Thanks to the decoupling of the thermal and autogenous strains, as explained previously, the coefficient of thermal expansion was determined. The results can be seen in Figure 14a,b, which shows the CTE values as a function of equivalent age and degree of reaction, respectively. As mentioned previously, CTE progresses in four distinct stages. However, the first obtained value corresponds already to the end of the second stage, where the recorded minimum corresponds to the CTE of the solid skeleton. The initial phase during which a very high peak is obtained due to the paste being in a plastic phase and the contribution of water [11], is missed for AAS pastes because the reaction is faster than for OPC, as can be seen from the isothermal calorimetry results. In that stage, all compositions exhibit a CTE of around 10 µm/m/°C which is similar to OPC, before increasing to higher values between 33 and 55 µm/m/°C, while the CTE of OPC only reaches between 20 and 25 µm/m/°C).

When examining the results after 200 h, it can be observed that increasing the S/B ratio results in a higher stabilization value for the CTE of the compositions. In fact, the CTE of the solution is higher than that of the solid components. Moreover, a higher S/B involves the formation of more pores which can result in higher pressure as a consequence of temperature changes [71]. And because the E-modulus is lower in this case [72], the material exhibits higher strains. Additionally, an increase in the S/B ratio slows down the evolution and, as a result, the stabilization of the CTE.

An increase in the concentration leads to an increase in CTE. When the concentration is equal to 0.5 M and 2 M, an increase in the concentration accelerates the evolution of the CTE. An interesting composition to examine in terms of CTE results is S08M8, as it exhibits a unique behavior compared with the other pastes. The CTE of S08M8 increases very slowly over time and with the degree of reaction, but ultimately, it reaches a significantly higher value than the other pastes. In contrast, in OPC, the increase in CTE is typically due to a decrease in the IRH in the paste depending on the hydration process and on the environmental conditions if the material is not sealed, which is not the case in the present research.

According to Canciam (2014) [73], the (volumetric) CTE of sodium hydroxide ranges between 4.71 and 5.07 · 10^−4^/°C for weight ratio comprised between 1% and 50% (which includes the studied concentrations). This corresponds to a linear CTE between 157 and 169 µm/m/°C because the solution is homogeneous and isotropic; consequently, the volumetric CTE can be divided by 3 to obtain the linear one. In comparison, the volumetric CTE of water is 2.07 · 10^−4^/°C, which corresponds to a linear CTE of 69 µm/m/°C. On a longer-term basis, the CTE of AAS is higher than that of OPC paste. This difference can be attributed to the higher CTE of the alkaline solution, which is 2.27 to 2.45 times higher than that of water. Except for S08M8, the CTE of AAS also tends to develop earlier.

### 4.7. Internal Relative Humidity

**Figure 15:**
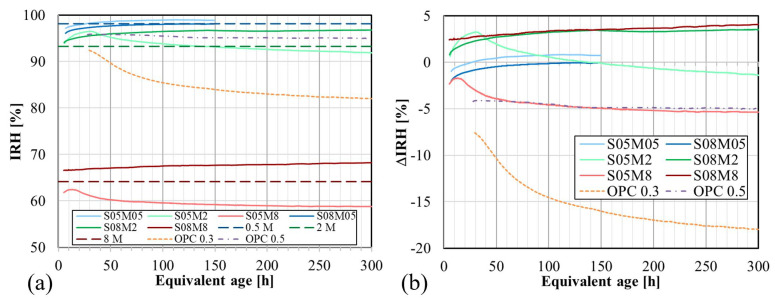
Internal relative humidity of AAS pastes: (**a**) IRH as a function of the age; (**b**) IRH with respect to RH of the solution as a function of age.

Internal relative humidity (IRH) plays a crucial role in all types of deformation, including autogenous deformation. When concrete absorbs water, the IRH increases due to the greater amount of water available. The internal relative humidity as a function of the age for the different compositions can be found in Figure 15a. The relative humidity (RH) of the alkaline solutions was also measured and is shown in the same figures as 0.5 M, 2 M, and 8 M. The RH of the solutions was subtracted from the IRH (ΔIRH) because it is assumed that the initial IRH of the paste is equal to the RH of the used solution. These results are presented in Figure 15b. The monitoring of S05M05 and S08M05 was stopped earlier than for the other compositions due to the magnitude of the results (≥98%), which could potentially damage the sensors.

For two different S/B ratios, the behavior of IRH varies significantly for higher concentrations (≥2 M). At a low S/B ratio (and ≥2 M), there is initially an increase in the IRH, followed by a decrease at around 31 h for S05M2 and 13 h for S05M8. This increase is likely linked to the swelling of the paste, as materials tend to swell when the IRH increases. Similarly, the decrease is likely linked to a reduction in volume due to the self-desiccation shrinkage. Equation (6) [74] is the Kelvin–Laplace equation, which links the pore pressure of fluid to IRH. When the binder is hydrated, the IRH decreases over time due to the consumption of internal solution during the reaction. This results in negative pore pressure, causing contraction of the solution molecules on the meniscus, leading to self-desiccation [74]. Hu et al. [75] explain that in the case of alkali-activated fly ash, the measured IRH starts at a low value and increases, unlike OPC which starts very high and only decreases. These values are, however, similar to the relative humidity of the alkali activators. The high ion concentration present in the pore solution is linked to the alkalinity of the activator and to the ions dissolved from the fly ash. It is explained that in OPC, the decrease in IRH is directly linked to the hydration time due to self-desiccation. During the reaction of AAM, ions present in the solution combine with the reaction products, leading to a decrease in the total ion concentration of the solution, bringing about the development of the IRH. In contrast, at high S/B, the IRH only increases, indicating ongoing swelling. Lastly, it can be seen that S05M05 and S08M05 only exhibit an increase in the IRH.

(6) $$p^{\prime }=\frac{-ln(IRH)\cdot R\cdot T}{v^{\prime }}$$

where the following notation is used:

- *p*′: pore pressure of fluid;
- *IRH*: internal relative humidity;
- *R*: universal gas constant;
- *T*: absolute temperature;
- *v*′: molar volume of solution.

Regarding the alkaline concentration, the measured IRH for S05M8 and S08M8 is significantly lower than that of the other compositions. As previously mentioned, the RH of the solution swas also measured, revealing that the results for each composition fall in the range of the RH of the used solution. The RH of the solution is 64% for 8 M, 93% for 2 M, and 98% for 0.5 M. The concentration has a direct effect of reducing the RH of the solution and thus the IRH of the pastes. At low S/B, reducing the concentration appears to delay the decrease in IRH over time. When examining the degree of reaction, the turning point seems to occur earlier for S05M8 than for S05M2. This observation suggests that the reaction in S05M8 is faster and larger than in S05M2, as evidenced by the isothermal calorimetry results, see Section 4.4.

The effect of S/B on IRH in AAS is similar to that observed in OPC pastes. However, the resulting decrease in IRH with decreasing S/B ratio is much smaller for AAS than for OPC. Studies by Yssorche-Cubaynes et al. [76] have demonstrated that OPC pastes with W/C between 0.27 and 0.75 maintain an IRH above 75% throughout the entire experiment. In contrast, for AAS pastes, as the concentration of the alkaline solution is reduced, the IRH tends to be closer to the range observed in OPC pastes, which is equivalent to having a solution with a concentration of 0 M.

**Figure 16:**
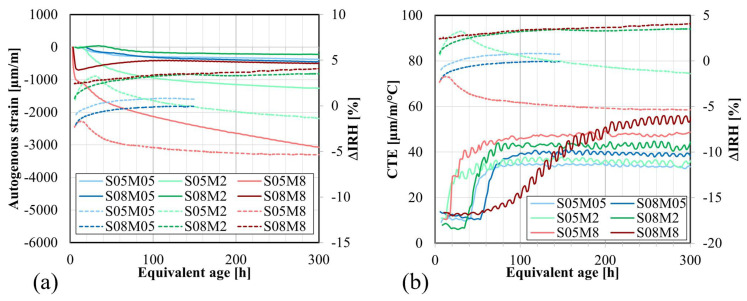
Comparison of the IRH for AAS pastes with (**a**) autogenous strain; (**b**) coefficient of thermal expansion (Legend: — autogenous strain or CTE; - - - IRH).

Figure 16a compares the autogenous strain and the ΔIRH of the different compositions, as functions of the equivalent age. For compositions such as S05M8 and S05M2, characterized by significant self-desiccation shrinkage, there is a significant decrease in the IRH over time. This decrease in IRH is concurrent with the occurrence of high self-desiccation shrinkage [25]. On the other hand, swelling is linked to an increase in the IRH (for S08M8) or only a small decrease in the IRH as for S08M2, S08M05, and S05M05.

Figure 16b presents a comparison between the CTE and the ΔIRH for the different compositions. In OPC pastes, the CTE is influenced by the internal relative humidity of the paste, with a decrease in IRH resulting in an increase in CTE if the IRH is higher than 60% [11,77]. As seen previously, the RH of the used solutions increased when the concentration decreased. The CTE is higher when the concentration of the solution is higher because the RH of the solution is lower. For S05M2 and S05M8, in which desiccation is observed in the IRH results, there is an increase in CTE followed by stabilization, while the IRH decreases. However, when comparing the impact of S/B, the assumption is no longer valid, as a higher S/B ratio leads to an increase in both IRH and CTE. Therefore, the development of CTE is not solely determined by the evolution of the IRH for AAM. It should be noted that the conclusions drawn are based on sealed conditions. In scenarios where the material is unsealed and the IRH has reached a stable state, any environmental changes in RH could still result in a similar effect on the CTE as observed in OPC.

**Figure 17:**
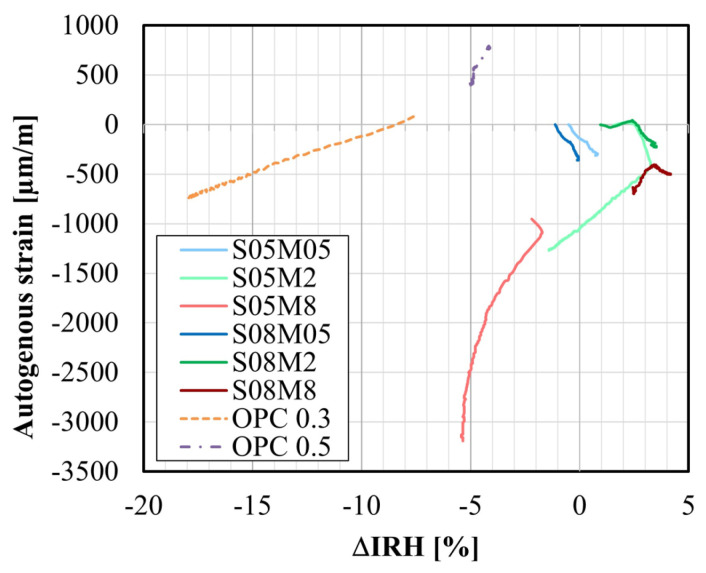
Autogenous strain as a function of the IRH variation (ΔIRH).

Figure 17 displays the autogenous strain as a function of ΔIRH. The relation between the two parameters is linear for OPC pastes, where an increase in IRH results in an increase in autogenous strain. Conversely, a linear correlation is also noticeable for S05M05 and S08M05. However, this time, an increase in IRH leads to a decrease in autogenous strain. For the other composition, a bump is observable. When the S/B is equal to 0.5, the turning point seems to be the IRH while for S/B = 0.8, it appears to be the autogenous strain because the IRH continues to increase during the evolution of the autogenous strain.

## 5. Conclusions

Based on the experimental findings, several conclusions can be drawn regarding the evolution of the early-age volume changes in AAS pastes:

1. The autogenous swelling is powered by the increase in the S/B ratio and the increase in the molarity, while the self-desiccation shrinkage is decreased by increasing S/B or decreasing the concentration of the alkaline solution.
2. The CTE of AAS (33–55 µm/m/°C) is notably higher than that of OPC (20–25 µm/m/°C) due to the elevated CTE of the solution (2.27 to 2.45 times higher than the CTE of water), implying that the thermal strains in AAS will be higher than in OPC. A higher S/B leads to a higher CTE due to the addition of solution, which has a higher CTE than solids. It also slows down the evolution of the CTE. Similarly, an increase in the concentration enhances the CTE and accelerates its evolution.
3. The IRH primarily depends on the concentration of the alkaline solution. Increasing the concentration leads to a decreased IRH, amounting to about 64% when using an 8 M solution. In compositions with S/B = 0.5, IRH initially increases because of the decrease in the total ion concentration before decreasing due to self-desiccation. Conversely, at S/B = 0.8, only an increase in IRH is observed, correlating with higher swelling and reduced self-desiccation observed in the autogenous strain. The opposite trend established between the IRH and the CTE for OPC was observed when examining the effect of the concentration. However, this was not fully consistent with the effect of S/B.
4. Vicat and isothermal calorimetry methods demonstrate good agreement for the determination of the setting times, for lower S/B and higher concentrations. However, the knee-point method presents challenges for compositions lacking apparent swelling. Generally, increasing S/B prolongs setting times due to the addition of water to the paste, while higher concentration accelerates setting times by promoting polymerization.
5. The heat flow and the cumulative heat are predominantly influenced by the concentration of the alkaline solution due to the higher availability of OH^−^. Over time, a higher S/B results in increased heat release. Extrapolation revealed an optimum ultimate heat for an alkali content of 3.57%. Increasing S/B and the concentration generally enhanced the ultimate heat. The average apparent activation energy of 75.5 kJ/mol was computed.
6. Enhanced workability is observed with increasing S/B and concentration due to the addition of solution and higher particle dispersion. The compressive strength increases with the increase in the concentration and the decrease in S/B, attributed to the formation of the strength-giving gel and the reduction in the pores.

In this research, the tests were conducted at 20 °C. Changing the curing temperature can also affect the studied properties. Moreover, concerning the autogenous strain, the next step is to decouple the swelling and the self-desiccation shrinkage as both overlap for some compositions. This would allow an even better understanding of the phenomenon. Finally, a microscopic study, including pore solution or reaction products analysis, on the material could improve the investigation. In addition, combining the autogenous strains with drying strain, coupled with different temperature histories, would also expand the understanding of these materials.

## Figures and Tables

**Table 1:** Chemical composition [%] of BFS in mass percent from X-ray fluorescence spectroscopy [40].

SiO_2_	Al_2_O_3_	Fe_2_O_3_	CaO	K_2_O	MgO	TiO_2_	SO_3_	Na_2_O	BaO	MnO
33.30	12.30	0.39	40.80	0.67	7.84	1.29	2.30	0.44	0.31	0.36

**Table 2:** Mix proportions of the pastes.

Paste	S/B [-]	BFS [g]	NaOH 0.5 M [g]	NaOH 2 M [g]	NaOH 8 M [g]
S05M05	0.5	100	50	0	0
S05M2	0.5	100	0	50	0
S05M8	0.5	100	0	0	50
S08M05	0.8	100	80	0	0
S08M2	0.8	100	0	80	0
S08M8	0.8	100	0	0	80

**Table 3:** Ultimate heat release of each AAS paste.

Composition	Q_∞_ [J/g]	R^2^ [-]	Extrapolation Interval [h]
S05M05	75.07	0.9920	15.5–800.0
S05M2	210.67	0.9993	13.0–190.0
S05M8	321.37	0.9972	8.0–190.0
S08M05	76.72	0.9985	18.0–200.0
S08M2	262.80	1.0000	18.0–500.0
S08M8	376.97	0.9982	18.0–333.0

**Table 4:** Setting times of AAS pastes.

Composition	IS (Vicat) [h]	FS (Vicat) [h]	IS (IsoCal) [h]	FS (IsoCal) [h]	Diff IS [h]	Diff FS [h]
S05M05	11.68	13.08	2.76	13.14	8.92	0.06
S05M2	3.08	4.05	1.31	4.78	1.78	0.73
S05M8	0.90	2.72	1.06	1.74	0.16	0.98
S08M05	15.40	19.10	2.04	14.24	13.36	4.86
S08M2	4.48	5.85	1.44	4.57	3.04	1.28
S08M8	2.03	3.33	1.07	2.03	0.96	1.30

**Table 5:** Apparent activation energy of each composition.

Composition	S05M05	S05M2	S05M8	S08M05	S08M2	S08M8	Average
E_a_	74.18	71.99	83.63	69.08	73.08	81.05	75.50

**Table 6:** Setting times of AAS pastes with knee-point method.

Composition	FS (Vicat) [h]	FS (IsoCal) [h]	FS Knee-Point Method [h]
S05M05	13.08	13.14	35.58
S05M2	4.05	4.78	5.85
S05M8	2.72	1.74	12.28
S08M05	19.10	14.24	49.03
S08M2	5.85	4.57	8.75
S08M8	3.33	2.03	9.17

## Data Availability

Data is contained within the article.

## Abbreviations

The following abbreviations are used in this manuscript:

AAM	Alkali-activated materials
AAS	Alkali-activated slag
CTE	Coefficient of thermal expansion
IRH	Internal relative humidity
M	Mol/l
OPC	Ordinary Portland cement
RH	Relative humidity
S/B	Solution-to-binder mass ratio
W/C	Water-to-cement mass ratio
